# Clinical and radiological outcomes of long COVID-19 and post-COVID fibrosis: Correspondence

**DOI:** 10.5578/tt.20239922

**Published:** 2023-06-13

**Authors:** Rujittika Mungmunpuntipantip, Viroj Wiwanitkit

**Affiliations:** 1 Consultant, Private Academic Consultant, Bangkok, Thailand; 2 Research Center, Chandigarh University, Punjab, India

Dear Editor

Dear Editor, we would like to share ideas on the publication “Clinical and radiological outcomes of long COVID: Is the postCOVID fibrosis common? ( 1 ).” In the third month following COVID-19, Sarolu et al. sought to study clinical and functional evaluation as well as radiologic alterations ( 1 ). Dyspnea was the most prevalent symptom and fibrotic-like alterations and GGO were the most prevalent radiological findings in the third month after COVID-19, according to Sarolu et al. ( 1 ). Longer follow-up investigations of COVID-19 survivors are required, according to Sarolu et al. ( 1 ), to detect long-lasting alterations.

Long-COVID-19 has a problem that has to be fixed immediately. There are still important problems that require addressing. The patient’s original, apparent clinical diagnosis was supported by COVID-19, even if there is still a probability that the patient has unidentified co-morbid conditions. Moreover, the patient may receive a second COVID-19 ( 2 ). It’s crucial to discuss previous vaccinations as well. There must be enough information to establish a conclusion on how the disease affects health issues. There should be sufficient knowledge. It is conceivable that protracted COVID, also known as extended COVID-19, is not the primary reason why a patient with an illness develops such symptoms.

## References

[bb0005] Sarıoğlu N,  Aksu  GD,  Çoban   H,  Bülbül   E,  Demirpolat  G,  Arslan   AT (2023). Clinical and radiological outcomes of long
COVID: Is the post-COVID fibrosis common? Tuberk
Toraks 2023.

[bb0010] Haq ZU, Fazid  S,  Yousafzai YM,  Noor M, Ibrahimzai AK,  Iqbal A (2021). Repeated COVID-19 infection exists: A case
series from Pakistan. J Ayub Med Coll Abbottabad 2021.

